# Purification of murine cortex mitochondria using a modified magnetic bead isolation method^☆^

**DOI:** 10.1016/j.mex.2026.103871

**Published:** 2026-03-18

**Authors:** Eva Josic, Ana Ujevic, Borna Puljko, Nikolina Maček Hrvat, Adriana Lipovcic, Svjetlana Kalanj-Bognar, Kristina Mlinac-Jerkovic

**Affiliations:** a Laboratory for Molecular Neurobiology and Neurochemistry, Croatian Institute for Brain Research and Department of Chemistry and Biochemistry, School of Medicine, University of Zagreb, Zagreb, Croatia; b Institute for Medical Research and Occupational Health, Zagreb, Croatia

**Keywords:** Plasma membrane contamination, Mitochondria viability, Subcellular fractionation, Magnetic beads purification

## Abstract

We describe a method for purifying murine cortex mitochondria, based on a modification of available technology. The mitochondria isolated and purified using this modified method are viable, without plasma membrane contamination, and suitable for downstream analyses. The method is based on magnetic-activated cell sorting (MACS) technology: extracting mitochondria using a commercially available kit, followed by isolation of mitochondria using antibody-labeled magnetic beads and additional washing steps. We identify the steps crucial for purification from plasma membrane contamination, including optimal starting tissue weight, essential reagents and their preparation, as well as other experimental conditions. The viability of purified mitochondria is assessed using fluorescent imaging. Furthermore, we compare the purity using two additional suboptimal methods in regards to plasma membrane contamination: one that employs an additional commercial kit and protocol, and another that is based on differential centrifugation. The method of choice results in a lower mitochondrial yield, but maximal purity in respect to copurification with other cellular membranes, which is especially important for downstream analyses of high sensitivity.

• Successfully overcoming the notoriously challenging step of purification mitochondria free from plasma membrane contamination.

• Purity is also achieved in respect to other cellular organelle contamination.

• Viable mitochondria is obtained, suitable for downstream analyses.

## Graphical abstract

**Figure:**
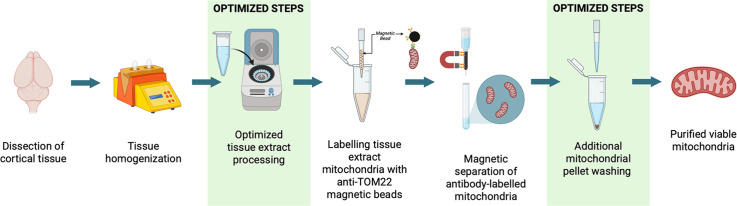

**Specifications table**

**Table:** 

**Subject area**	Biochemistry, Genetics and Molecular Biology
**More specific subject area**	Subcellular fractionation
**Name of your method**	Mitochondria purification
**Name and reference of original method**	
**Resource availability**	Mitochondria Extraction Kit - Tissue | Miltenyi Biotec Mitochondria Isolation Kit, mouse tissue | Miltenyi Biotec gentleMACS™ Dissociator | Miltenyi Biotec MACS® MultiStand | Miltenyi Biotec LS Columns | Miltenyi Biotec

## Background

Purification of mitochondria for subsequent functional and structural studies is traditionally carried out using methods based on differential centrifugation or, more recently, commercial kits based on magnetic purification. Normally, the tissue is homogenized, and the plasma membrane is either mechanically ruptured or lysed with various chemical reagents. The lysed homogenate is further fractionated by differential centrifugation or using magnetic antibody-labelled beads that recognize specific mitochondrial proteins and bind intact mitochondria, which can then be separated and purified from other organelles and contaminants on a magnetic column [1,2]. One such commercially available workflow is used here, described as an optimal method, alongside a second method that uses additional steps and commercial kits, and a third method previously published by Mukherjee et al., which is based on differential centrifugation [3]. With many published protocols and methods using cultured cells or non-neural metabolically active tissues as starting materials, the literature on purification from myelin-rich brain tissues is scarce. Notably, what almost every published protocol lacks, whether based on differential centrifugation or affinity purification, is proof of purification from the plasma membrane contaminants, that is crucial for studies aiming to examine the mitochondrial lipidome [1,[4], [5], [6], [7], [8], [9], [10], [11], [12]]. This only accentuates the need to further optimize purification protocols, enabling better quality control of the studied mitochondrial samples. While most downstream functional analyses don´t require optimal mitochondrial purity in respect to the plasma membrane contamination, structural and compositional studies do.

## Method details

### Materials

#### Animals and tissues

A total of 28 adult mice of both sexes, aged 4–6 months and with the same genetic background (C57BL/6), were used, 18 of which were used for experiments in this study, and 10 were used for preliminary experiments (data not shown). The animals were group-housed, kept under standardized temperature and humidity and a 12 h light-dark cycle with water and food ad libitum in standard cages. For all experiments, the mice were anesthetized with isoflurane (Vetopharma Animal Health, S.L., Barcelona, Spain) overdose until loss of consciousness, euthanized by cervical dislocation and decapitated. Brains were rapidly removed, dissected, and kept at −20 °C until use. All experimental procedures were performed in accordance with the ARRIVE guidelines [13]. All procedures were approved by jurisdictional ethics committees for scientific experiments and approved by the appropriate institutions in accordance with institutional and government guidelines (Number 251-59-10,106-24-111/169; class 641–01/24–02/04 for the project NEUROGEM to K.M.-J.).

#### Reagents

Mitochondria Extraction Kit – Tissue (Miltenyi Biotec, Cat#130-097-340)

Mitochondria Isolation Kit, mouse tissue (Miltenyi Biotec, Cat#130-096-946)

Myelin Removal Beads II, human, mouse, rat (Miltenyi Biotec, Cat#130-096-433)

Dulbecco’s PBS (1X), without Ca and Mg, without Phenol Red (Capricorn Scientific, Cat#PBS-1A)

Roche cOmplete™, Mini, EDTA-free Protease Inhibitor Cocktail (Roche, Cat#11836170001)

Bovine Serum Albumin heat shock fraction, pH 7, ≥ 98 % (Sigma-Aldrich, Cat#A7906)

BioTracker 633 Red Mitochondria Dye (Sigma-Aldrich, Cat#SCT137)

SuperSignal™ West Femto Maximum Sensitivity Substrate (Thermo Scientific, Cat#34096)

Pierce™ BCA Protein Assay Kit (Thermo Scientific, Cat#23227)

#### Antibodies

##### Primary antibodies

anti-TOM20 (Translocase of outer mitochondrial membrane 20 homolog), rabbit recombinant monoclonal antibody (Proteintech, Cat#80501-1-RR), dilution 1:5000

anti-PMCA (Plasma membrane-type Ca^2+^-ATPase), mouse monoclonal antibody (Santa Cruz Biotechnology, Cat#SC-271917), dilution 1:1000

anti-TfR (Human transferrin receptor), mouse polyclonal antibody (Thermo Fisher, Cat#13–6800), dilution 1:2000

anti-Thy1 (Thymus cell antigen 1, theta), rat polyclonal antibody (BD Biosciences, Cat#553000), dilution 1:1000

anti-SIGMAR1 (Sigma non-opioid intracellular receptor 1), rabbit polyclonal antibody (Proteintech, Cat#15168-1-AP), dilution 1:4000

anti-PDHA2 (Pyruvate dehydrogenase (lipoamide) alpha 2), rabbit polyclonal antibody (Proteintech, Cat#17134-1-AP), dilution 1:3000

anti-PEX3 (Peroxisomal biogenesis factor 3), rabbit polyclonal antibody (Proteintech, Cat#30,424-1-AP), dilution 1:750

anti-CALR (Calreticulin), rabbit polyclonal antibody (Proteintech, Cat#10,292-1-AP), dilution 1:1250

##### Secondary antibodies

Peroxidase AffiniPure® Goat Anti-Rabbit IgG (*H* + *L*) (Jackson Immunoresearch, Cat#111–035–003), dilution 1:50 000

Peroxidase AffiniPure® Donkey Anti-Mouse IgG (*H* + *L*) (Jackson Immunoresearch, Cat#715–035–150), dilution 1:50 000

Peroxidase AffiniPure® Donkey Anti-Rat IgG (*H* + *L*) (Jackson Immunoresearch, Cat#712–035–153), dilution 1:50 000

Alexa Fluor® 488 AffiniPure® Goat Anti-Rabbit IgG (*H* + *L*) (Jackson Immunoresearch, Cat#111–545–003), dilution 1:400

#### Equipment

Potter-Elvehjem tissue homogenizer (Bellco Glass, Inc., Cat#50–194–5203) gentleMACS™ Dissociator (Miltenyi Biotec, Cat#130–093–235) gentleMACS™ C Tubes (Miltenyi Biotec, Cat#130–093–237)

MidiMACS™ Separator (Miltenyi Biotec, Cat#130–042–302)

MACS® MultiStand (Miltenyi Biotec, Cat#130–042–303)

LS Columns (Miltenyi Biotec, Cat#130–042–401)

Pre-Separation Filters (30 µm) (Miltenyi Biotec, Cat#130–041–407)

Hettich Universal 32 R Centrifuge

Biorad ChemiDoc™ MP Imaging System

Olympus FV3000 confocal microscope

### Procedure

#### Buffer preparation

Prepare all buffers fresh for the isolation procedure and keep them on ice.

#### 100X Lysis buffer

100X Lysis buffer at pH 7.8 (350 mM Tris-HCl (Sigma-Aldrich, Cat#252859), 0.2 M NaCl (T.T.T., Cat#7647–14–5), 50 mM (Mg(CH_3_COO)_2_ x 4H_2_O) (Sigma Aldrich, Cat#M5661).

Weigh the salts and dissolve them in double-distilled water (ddH₂O) to about 90% of the total buffer volume. Adjust the pH using concentrated HCl and add ddH₂O to the final buffer volume. To prepare Lysis buffer, dilute 1:100 the prepared 100X Lysis Buffer to the desired volume using ddH₂O. Required volume per sample weighing 50 mg is 2 mL of Lysis Buffer and 100 µL of 100X Lysis Buffer.

#### Solution 2 (Part of Mitochondria Extraction Kit – Tissue (Miltenyi Biotec, Cat#130–097–340))

Prepare the 1X Solution 2 by adding 1 vol of 10X Solution 2 to 9 vol of ddH₂O. Required volume per sample weighing 50 mg is 5 mL, and for preparing the Protease Inhibition Buffer, additional 10 mL per Protease Inhibitor tablet.

#### Extraction buffer

Prepare the Extraction Buffer by adding 20 µL of Solution 1 (Part of Mitochondria Extraction Kit – Tissue (Miltenyi Biotec, Cat#130–097–340)) to 0.5 mL of freshly prepared 1X Solution 2. Required volume per sample weighing 50 mg is 500 µL.

#### Protease inhibition buffer

Prepare the Protease Inhibition Buffer by dissolving one protease inhibitor tablet in the freshly prepared 1X Solution 2, according to the Mitochondria Extraction Kit – Tissue (Miltenyi Biotec, Cat#130–097–340) protocol. Required volume per sample weighing 50 mg is 2 mL.

#### Myelin removal buffer

Prepare the 0.5 % Buffer by dissolving Bovine serum albumin in sterile PBS (without calcium and magnesium) and adjust pH (if needed) to 7.2. Required volume per sample weighing 50 mg is 10 mL.

#### Separation Buffer (Part of Mitochondria Isolation Kit, mouse tissue (Miltenyi Biotec, Cat#130–096–946))

Prepare the 1X Separation Buffer by first incubating 10X Separation Buffer at 37 °C until visible crystals are no longer present, and dilute to 1X by mixing 1 part of 10X Separation Buffer and 9 parts ddH₂O. Required volume per sample weighing 50 mg is 23 mL.

#### Isolation procedures

##### Method 1

This method is based on Mitochondria Extraction Kit – Tissue and Mitochondria Isolation Kit mouse tissue, and their respective protocols by Miltenyi Biotec, with adjusted centrifugation conditions and added washing steps on final purified fractions. Steps in the described method below differing from the Miltenyi Biotec protocols are marked with *.

1. All reagents, samples and buffers were kept on ice.
2. Weigh murine cortex tissue at about 50 mg ± 5 mg.
3. *Wash tissue in an Eppendorf tube by adding 0.5 mL of Solution 2 3 × 5 min on ice. Discard Solution 2 using a pipette after each wash.
4. *Add 0.5 mL of Extraction buffer to the tissue and, using a Potter-Elvehjem pestle, perform a gentle and slow homogenisation movement by pressing the tissue against the tube wall and rotating the pestle, for 3 strokes.
5. Incubate for 30 min on ice.
6. Centrifuge for 5 min at 300 g and 4 °C. Discard the supernatant completely.
7. *Add 1 mL of cold Protease Inhibition Buffer, resuspend the pellet by slowly pipetting, and transfer the resuspended pellet to a cold gentleMACS C tube. Wash the Eppendorf tube by adding 1 mL of cold Protease Inhibition Buffer and transfer the Buffer to the C tube.
8. Perform homogenisation on gentleMACS Dissociator with program m_mito_tissue_01.
9. *Centrifuge the homogenate in the C-tubes right after homogenisation, at 500 g for 10 min at 4 °C.
10. *Remove the supernatant carefully by not disrupting the pellet, and leave about 200–300 µL of supernatant at the bottom of the tube. In this step the pellet is still very loose - avoid aspirating it with the supernatant. Collect the supernatant.
11. Add the cooled 1X Separation Buffer to the supernatant to a final volume of 10 mL and mix well.
12. Add 50 µL of Anti-TOM22 beads to the supernatant diluted in 1X Separation Buffer.
13. *Incubate for 1 h at 4 °C with continuous gentle vortexing (no faster than 20 rpm on a tube rotator).
14. Prepare the LS column by placing it on MidiMACS Separator positioned on the MACS Multistand and rinsing with 3 mL of 1X Separation Buffer.
15. Place the 30 µm Pre-Separation Filter on the column and add the incubated sample in batches of 3 × 3.3 mL.
16. Once the flow-through is completely eluted from the column, wash the filter and the column by adding 3 × 3 mL of Separation Buffer. Let the wash flow through completely before adding the next volume of Separation Buffer.
17. Discard the filter, remove the column from the separator and place it above an Eppendorf tube.
18. Add 1.5 mL of Separation Buffer to the column and firmly press the plunger until the sample is eluted. Immediately proceed to the next step.
19. Centrifuge the eluted mitochondrial fraction at 13 000 g for 2 min at 4 °C. Discard the supernatant completely.
20. * Wash the pellet by carefully adding 500 µL of sterile PBS (Ca and Mg free), optimally by not disrupting the pellet. Remove the supernatant right away. Centrifuge if needed.
  a. If the pellet is disrupted, repeat the centrifugation at conditions from step 19. Remove the supernatant completely.
21. * Repeat step 20. (or 20. a.).
22. Resuspend the mitochondrial pellet in 100 µL of the Storage Buffer.

##### Method 2

This method differs from the above-described optimal workflow in an additional step of myelin removal, using the Myelin Removal Beads II, human, mouse, rat (Miltenyi Biotec, Cat#130–096–433). The added steps described below are performed from step 10 of the above-described Method 1 (the first nine steps are identical). Steps in the described method below differing from the Miltenyi Biotec protocols are marked with *.

10.1. Add Myelin Removal Buffer and Myelin Removal Beads II, human, mouse, rat in amounts according to the starting tissue weight as recommended in Myelin Removal Beads II human, mouse, rat data sheet (20 µL of Myelin removal beads and 180 µL of Myelin Removal Buffer per 50 mg starting sample weight).

10.2. Mix the samples by gently mixing without vortexing. Incubate in the refrigerator at 4 °C for 15 min.

10.3. Wash the mitochondria by adding 10X the Myelin Removal Buffer volume added in step 10.1.

10.4. *Centrifuge at 1000 g for 15 min at 4 °C. Remove the supernatant completely.

10.5. Resuspend the supernatant completely by adding 1000 µL of Myelin Removal Buffer and pipetting.

10.6. Prepare the LS column by placing it on MidiMACS Separator positioned on the MACS Multistand and rinsing with 3 mL of 1X Myelin Removal Buffer.

10.7. Add the sample directly to the column and collect the flow-through.

10.8. Wash the column with 2 × 1 mL Myelin Removal Buffer and collect the total effluent with the flow-through from step 10.7.

11.1. *Alternatively to step 11. From the Method 1 protocol, add 1X cooled Separation Buffer to the pooled effluent collected in steps 10.7 and 10.8 to a final volume of 10 mL. Proceed with the second part of the Method 1 protocol as described above, from step 12.

##### Method 3

This method differs from the above-described Method 1 workflow in a lysis step – Instead of using the Mitochondria Extraction Kit – Tissue, lysis is performed as previously described in Mukherjee et al. [3], resulting in a purified mitochondrial pellet in the last centrifugation step. This final pellet is then further purified using the Mitochondria Isolation Kit mouse tissue, starting with Method 1, step 11.

1. Wash weighted samples in an Eppendorf tube by adding 0.5 mL of Lysis Buffer and removing immediately.
2. Incubate in 10% homogenate volume/mass of Lysis Buffer in an Eppendorf tube for 15 min at 4 °C
3. Homogenize using Potter-Elvehjem glass-teflon homogenizer with 10–15 strokes.
4. Add 1/9th volume of the homogenate (for 50 mg starting weight, that is about 50 µl) of 100X Lysis Buffer to the homogenate and centrifuge the homogenate at 3100 g for 5 min at 4 °C in a microcentrifuge.
5. Separate the pellet from the supernatant by pipetting.
6. Centrifuge the supernatant at 30,200 g for 5 min at 4 °C to separate the pellet of mitochondria from the post-mitochondrial or cytosolic fraction.
7. Add the cooled 1X Separation Buffer to the pellet to a final volume of 10 mL and mix well.
8. Add 50 µL of Anti-TOM22 beads to the pellet diluted in 1X Separation Buffer.
9. Proceed with the second part of the Method 1 protocol as described above, from step 13.

Notes

1. Prepare fresh buffers for each experiment.
2. Prepare the fresh Protease Inhibition Buffer immediately before use.
3. Optimal starting tissue weight is about 50 mg.
4. Best purity is obtained by first freezing the tissue at −20 °C, not using freshly dissected tissue for isolation, still yielding viable mitochondria.

## Method validation

All three methods are based on commercial Miltenyi kits with their proprietary protocols, with certain steps altered and optimized for maximal final purity, as highlighted in the method section (marked with an *). Final purified mitochondrial fractions were analyzed using Western blotting: LDS-PAGE (using Lithium dodecyl sulphate for protein denaturation instead of Sodium dodecyl sulphate in SDS-PAGE) followed by a transfer to PVDF membrane as previously described [14]. Post-transfer, membranes were tested for cellular markers, both organelle membranes and various plasma membrane markers. A low-femtogram sensitivity substrate (SuperSignal™ West Femto Maximum Sensitivity Substrate, Cat#34096) was used for immunodetection purposes. Purity was assessed according to the immunoreactivity of the plasma membrane marker and the mitochondrial marker on the Western blot PVDF membrane. The loading for LDS-PAGE was 10% of the final mitochondrial purified fraction. Namely, 10% of the final fraction was resolved on 4–12% PAGE gels (as described in the methods section, the final mitochondrial pellet was resuspended in 100 µL of Storage buffer), across all samples. This allowed for the comparison of final mitochondrial yield per method in the section Comparison of methods, considering the same starting cortical tissue mass for each isolation, as well as the assessment of sample purity. A detailed comparison of yield per method was not conducted, considering the very low reproducibility of suboptimal methods.

### Comparison of isolated mitochondria fraction purity between the methods

In each isolation, mitochondrial fractions were first immunodetected by Western blotting for panPMCA (plasma membrane marker, detecting all plasma membrane calcium ATPase isoforms) and TOM20 (mitochondrial marker, detecting translocase of outer mitochondrial membrane 20). To compare purities between and within the three methods, we used a ratio of chemiluminescent intensity of panPMCA marker to TOM20 marker in the final mitochondrial fraction calculated from ImageLab detected fluorescence intensities. Across all isolations, the Western blot protocol used for purity check was the same, with consistent electrophoresis, transfer and antibody incubation conditions. Each isolation method in this paper is represented in six replicates.

**Fig. 1:**
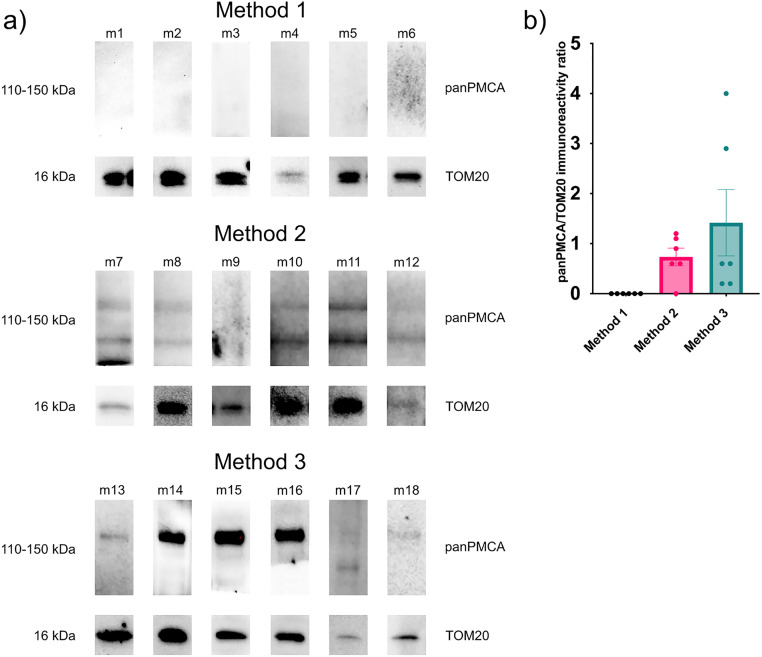
a) The purity of final isolated mitochondrial fractions compared by method, assessed by Western blotting against panPMCA, a plasma membrane marker, and TOM20, outer mitochondrial membrane marker. Presented are 6 isolations corresponding to 6 samples per method, with a total of 18 final purified mitochondrial fractions checked for purity a) low-femtogram sensitivity substrate was used for chemiluminescence visualization. b) Quantification of the final mitochondrial fraction purity using a ratio of panPMCA and TOM20 chemiluminescence intensity. Final protein yield was assessed using a BCA assay, with final concentrations: γ (m3) = 1014.36 µg/mL, γ (m4) = 808.64 µg/mL, γ (m5) = 88.36 µg/mL, γ (m6) = 41.82 µg/mL, γ (m7) = 730.36 µg/mL, γ (m8) = 341.18 µg/mL, γ (m9) = 73.09 µg/mL, γ (m10) = 73.18 µg/mL, γ (m11) = 184.55 µg/mL, γ (m12) = 70.91 µg/mL, γ (m13) = 329.18 µg/mL, γ (m14) = 1587.45 µg/mL, γ (m15) = 765.00 µg/mL, γ (m16) = 621.91 µg/mL, γ (m17) = 865.91 µg/mL, γ (m18) = 463.91 µg/mL. Samples m1 and m2 were below detection limit (20 µg/mL). Error bars shown the number of replicates ± SEM (standard error of mean).

As seen in Fig. 1, Method 1 consistently yields highly pure mitochondria in respect to the plasma membrane, with no PMCA isoforms detected across all repetitions. Method 3, based on a simple differential centrifugation method reported by Mukherjee et al. [3] and the Miltenyi Biotec Mitochondria Isolation kit, showed the lowest reproducibility, with PMCA still being highly abundant in most final fractions of isolated mitochondria. Method 2, also based on the commercial Miltenyi kit protocols as Method 1, showed higher reproducibility than Method 3 but still lower purity in comparison to Method 1. Furthermore, this method shows the lowest yield among the three methods, presumably due to the additional chromatography purification step. Method 1, being the simplest and yielding the highest mitochondrial purity, is the optimal of the three described methods.

### Confirmation of final fraction purity in respect to plasma membrane contamination and organelle contamination

To further confirm the purity of the final isolated mitochondrial fractions, the four representative fractions of purified mitochondria isolated using each of the methods were checked for additional plasma membrane markers and organelle markers. As plasma membrane markers, we used Thy1 and TfR, alongside panPMCA. The purity was also tested in relation to endoplasmic reticulum (ER) and peroxisomal contamination (detected by CALR and SigmaR1 for ER, and PEX2 for peroxisomes). To further assess the mitochondrial yield, we used PDHA2 antibody, which detects a subunit of pyruvate dehydrogenase, a mitochondrial matrix protein.

**Fig. 2:**
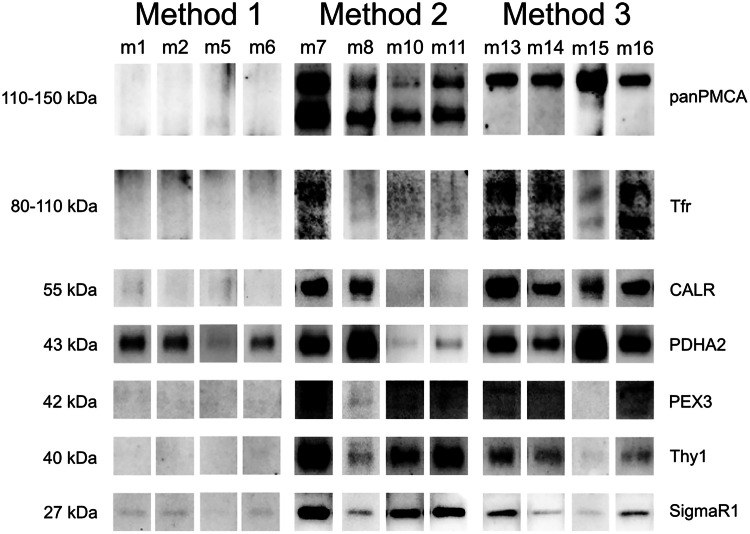
Comparison of mitochondrial purity between Method 1 and suboptimal methods, 2 and 3. All fractions are final purified mitochondrial fractions, resolved on 4-12% SDS-PAGE gel with purity assessed against the following markers: panPMCA (plasma membrane marker), TfR (plasma membrane marker), CALR (endoplasmic reticulum marker), PDHA2 (mitochondrial matrix marker), PEX3 (peroxisomal marker), Thy1 (plasma membrane marker), SigmaR1 (endoplasmic reticulum marker). A total of 12 isolated and purified samples is represented in the figure, with sample designations corresponding to Fig. 1 samples.

Both suboptimal methods lead to substantially lower purity, as seen in Fig. 2, with all three plasma membrane markers showing contamination present in the final purified mitochondria fractions. The same samples are contaminated with endoplasmic reticulum (ER), with visible Sigmar1 signals and strong Calreticulin signals. The samples isolated using Method 1 have lower mitochondrial yield, but also substantially higher purity in respect to the plasma membrane, with no visible plasma membrane marker signals (panPMCA, Thy1 and TfR). Additionally, endoplasmic reticulum markers Calreticulin and Sigmar1 show good purification of mitochondria in respect to the endoplasmic reticulum. Calreticulin is a soluble protein located in the ER lumen [15] and SigmaR1 is a transmembrane protein located in ER membranes, primarily in MAMs, but readily translocates to the ER lumen upon ER stress [16]. Calreticulin, being abundantly present in the suboptimal-method isolated mitochondria, and absent in the Method 1 isolated mitochondria, suggests the suboptimal methods copurify entire endoplasmic reticulum with mitochondria, whereas Method 1 successfully purifies mitochondria from ER. Lack of PEX3 signals shows efficient removal of peroxisomes across all samples and methods.

### MitoTracker imaging reveals viable isolated mitochondria

**Fig. 3:**
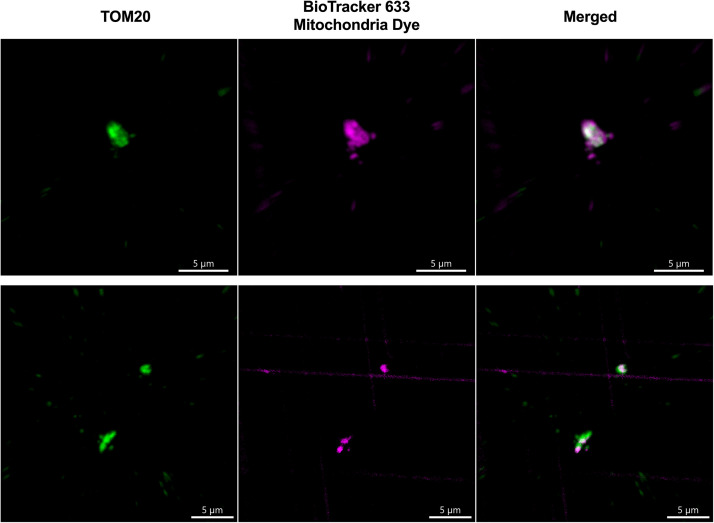
Final purified mitochondrial fractions stained with BioTracker 633 Red Mitochondria Dye (middle) and TOM20 (left) show viable pure mitochondria (merged; right). Scale bar is 5 µm.

In addition to achieving plasma membrane-free purification of mitochondria, Method 1 yields viable mitochondria. The viability of the final purified mitochondrial fractions was confirmed using the BioTracker 633 Red Mitochondria Dye, which specifically stains live mitochondria, and immunostaining using TOM20 as mitochondrial marker, detecting translocase of outer mitochondrial membrane 20 (Fig. 3). BioTracker 633 Red Mitochondria Dye-labelled and TOM20-labelled mitochondria were imaged using confocal microscopy.

Imaging additionally confirms the successful isolation of viable mitochondria using Method 1, and clear overlapping of both TOM20 signal and Biotracker 633 Red Mitochondria Dye signal. Mitochondria staining for fluorescent imaging was done by modifying published protocols [17,18].

## Limitations

None.

## Ethics statements

All experimental procedures complied with the ARRIVE guidelines and were carried out in accordance with EU Directive 2010/63/EU for animal experiments. All procedures were approved by regional ethics committees and by the appropriate institutions in accordance with institutional and government guidelines (Number 251–59–10,106-24–111/169; class 641–01/24–02/04 for the project NEUROGEM to K.M.-J.

## Credit author statement

**Eva Josic**: Methodology, Validation, Formal Analysis, Investigation, Writing – Original Draft, Writing - Review & Editing, Visualization. **Ana Ujevic**: Methodology, Validation, Investigation, Writing – Original Draft, Visualization. **Borna Puljko**: Conceptualization, Methodology, Validation, Investigation, Writing – Original Draft, Visualization. **Nikolina Maček Hrvat**: Methodology, Validation, Investigation, Writing – Original Draft. **Adriana Lipovcic**: Methodology, Validation, Investigation, Writing - Review & Editing. **Svjetlana Kalanj-Bognar**: Conceptualization, Resources, Writing - Original Draft, Writing - Review & Editing, Project Administration. **Kristina Mlinac-Jerkovic**: Conceptualization, Methodology, Investigation, Resources, Writing - Original Draft, Writing - Review & Editing, Supervision, Project Administration, Funding Acquisition. All authors have read and agreed to the published version of the manuscript.

Supplementary material *and/or* additional information [OPTIONAL].

## Declaration of competing interest

The authors declare that they have no known competing financial interests or personal relationships that could have appeared to influence the work reported in this paper.

## Data Availability

Data will be made available on request.
